# Supplementary material for: A Retrospective Analysis of Feeding Practices and Complications in Patients with Critical Bronchiolitis on Non-Invasive Respiratory Support
Source: Children (Basel). 2021 May 18;8(5):410. doi: 10.3390/children8050410 (PMC8157845; doi:10.3390/children8050410)
Supplement: Supplementary file 1 [file children-08-00410-s001.zip › children-1180016-supplementary.pdf]

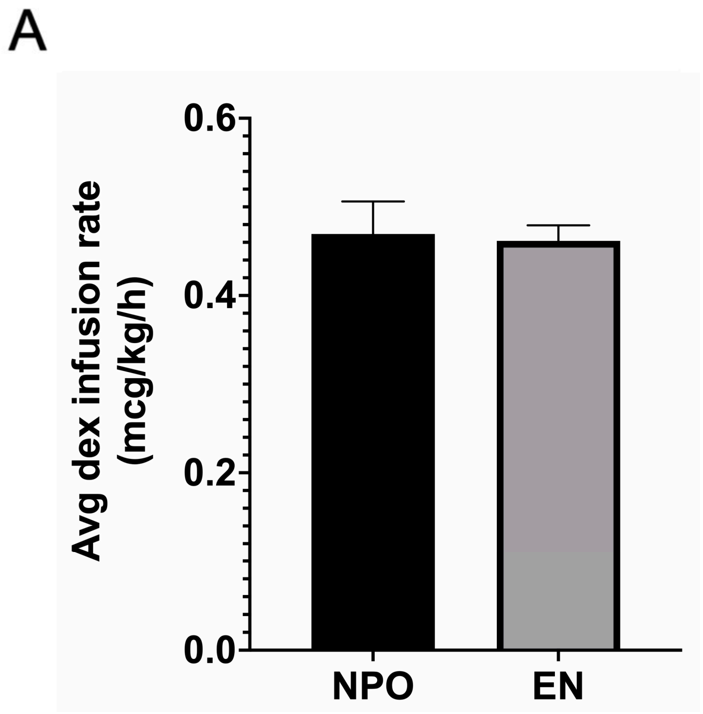

Dexmedetomidine	NPO	EN
**Prescribed**	28	62
**Not prescribed**	29	49

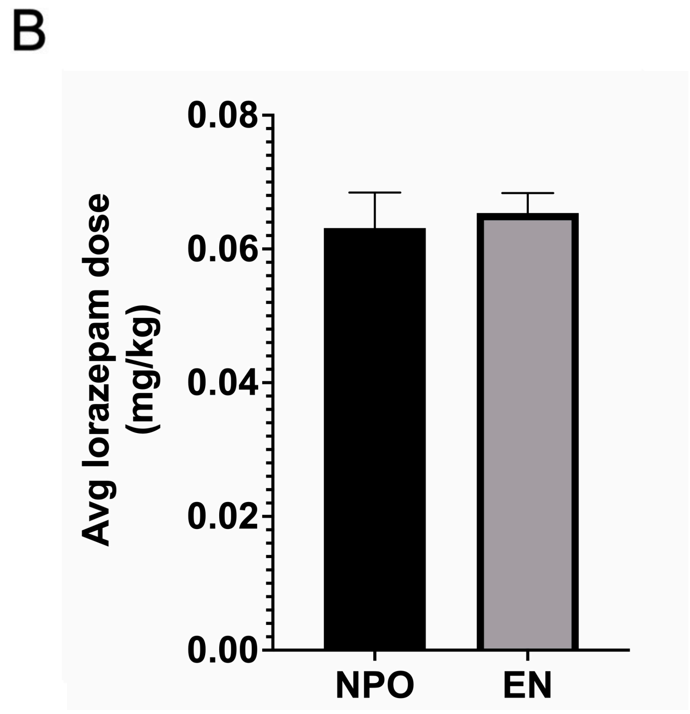

Lorazepam	NPO	EN
**Prescribed**	20	51
**Not prescribed**	37	60

Figure S1. Sedation data for the NPO and EN groups with respect to average doses of **(A)** dexmedetomidine and **(B)** lorazepam being administered to critically ill pediatric patients included in the study. Dexmedetomidine usage in NPO group was 51% and in the PO group was 57%. Lorazepam usage in NPO group was 36% and in PO group was 47%. Data presented as mean ± standard error of mean. Avg = average, dex = dexmedetomidine, NPO = nil per os (nothing by mouth), EN = enteral nutrition

Table S1. Demographic and Clinical Differences Between Groups (intubated only, *n* = 14)

	NPO (*n* = 9)	EN (*n* = 5)	*p*
Weight (kg), median (IQR)	7.7 (4.7-9.3)	5 (4.2-6)	0.15
Age (months), median (IQR)	6 (2.5-14)	3 (2-5)	0.25
PICU LOS (days), median (IQR)	11 (6-32.5)	15 (12.5-25.5)	0.38
NRS duration prior to intubation (days), median (IQR)	1.5 (1.1-2.3)	2.5 (1.9-4.5)	**0.044**
Intubation (hours after admission), median (IQR)	34 (28-68.5)	58 (42-130)	0.15
Intubation duration (days), median (IQR)	8 (3.5-22)	12 (4.5-15.5)	0.89
Pathogen (#), median (IQR)	3 (2-3.5)	3 (2-4)	0.83
Pathogen positive, *n* (%)	9 (100)	5 (100)	1.0
Single	1 (11)	0 (0)	0.44
Multiple	8 (89)	5 (100)	
Virus only	5 (55)	0 (0)	**0.038**
Virus + Bacteria	4 (45)	5 (100)	
Extubation failure, *n* (%)	1 (11)	1 (20)	0.65
Ventilator-free-days, median (IQR)	20 (6.5-24.5)	16 (12.5-23.5)	0.89
PRISM-III ROM, % (IQR)	0.8 (0.5-1.1)	0.5 (0.2-0.5)	0.09

IQR = interquartile range, PICU LOS = pediatric intensive care unit length of stay, NRS = non-invasive respiratory support, PRISM-III ROM = risk of mortality based on Pediatric Risk of Mortality-III score.
Wilcoxon rank-sum test used to compare continuous variables presented as median (interquartile range).
Chi-square test of independence used to compare categorical variables presented as *n* (%)

Table S2. Infecting Pathogen Characteristics (all groups, *n* = 178)

Pathogen Type	NPO (*n* = 64)	EN (*n* = 114)	*p**
Viral, *n* (%)			
Respiratory syncytial virus	35 (61)	71 (68)	0.12
Rhino/enterovirus	25 (44)	30 (29)	
Adenovirus	1 (2)	6 (6)	
Coronavirus	7 (12)	4 (4)	
Parainfluenza	4 (7)	6 (6)	
Human metapneumovirus	2 (4)	10 (10)	
Influenza A	0 (0)	1 (1)	
Bacterial, *n* (%)			
Streptococcus pneumoniae	2 (4)	2 (2)	0.51
Staphylococcus aureus	1 (2)	1 (1)	
Mycoplasma pneumoniae	0 (0)	1 (1)	
Moraxella catarrhalis	2 (4)	3 (3)	
Pseudomonas aeruginosa	1 (2)	0 (0)	
Hemophilus influenzae	5 (9)	2 (2)	
Presumed	7 (12)	14 (13)	

***** Chi-square test of independence used to compare categorical variables presented as *n* (%)

Table S3. Feeding Complications Encountered During Feeding Initiation (EN group only, *n* = 9)

List of Complications
1. Documented formula found in nose and mouth with bradycardia and desaturation requiring bagging.
2. Concern for respiratory status; apnea/bradycardia spells.
3. Emesis x2 afternoon prior to intubation; however, no reported aspiration.
4. Feeds stopped after 23 hours due to increased WOB, then restarted and increased slowly, reached goal while on HHF.
5. Feeds discontinued due to increased WOB and need for BiPAP.
6. NG feeds stopped due to increased WOB; restarted when clinically improved.
7. NG feeds started 4 hours after RAM but stopped due to increased WOB; reached full feeds once on HHF.
8. Patient did not tolerate bolus feeds, with multiple episodes of emesis, abdominal distention and hematemesis.
9. NG feeds started then paused due to increased WOB, then restarted and tolerated well.

WOB = work of breathing, HHF = heated high flow, BiPAP = bilevel positive airway pressure, NG = nasogastric
